# Effects of High-Concentrate-Induced SARA on Antioxidant Capacity, Immune Levels and Rumen Microbiota and Function in Goats

**DOI:** 10.3390/ani14020263

**Published:** 2024-01-15

**Authors:** Siqin Fan, Mengli Zheng, Ao Ren, Hongxiang Mao, Donglei Long, Lingyuan Yang

**Affiliations:** College of Animal Science and Technology, Hunan Agricultural University, Changsha 410125, China; fanjinyi0616@hunau.edu.cn (S.F.); ldl15073147518@foxmail.com (D.L.)

**Keywords:** goat, high concentrate, SARA, immunity, rumen, microbiota

## Abstract

**Simple Summary:**

Our study elucidated the specific alterations in rumen microbiota associated with subacute ruminal acidosis (SARA) triggered by a high-concentrate diet. It demonstrated that SARA can lead to a decrease in microbial richness and diversity in the rumen of goats. SARA could potentially hinder the growth of rumen bacterial communities. Within the context of SARA, the antioxidant capacity of goats diminishes, while inflammatory cytokines increase, potentially raising the risk of health issues in growing goats. Understanding this is crucial for predicting changes in goat microbiota and improving the efficiency and well-being of ruminants through nutritional approaches such as dietary interventions.

**Abstract:**

This study aims to explore the antioxidant, immune, and enzyme metabolism aspects in goats experiencing subacute ruminal acidosis (SARA). Furthermore, we seek to elucidate the relationship between the symbiotic microbiota of goats and their metabolic function. Sixteen goats were equally divided into two groups and fed a normal-concentrate diet (NC, 55% concentrate) or a high-concentrate diet (HC, 90% concentrate) for five weeks. We found that the HC diet reduced the total antioxidant capacity (T-AOC) (*p* = 0.022) and increased interleukin-1β (IL-1β) (*p* = 0.015), interleukin-4 (IL-4) (*p* = 0.008) and interleukin-6 (IL-6) (*p* = 0.002) concentration of goats. Simultaneously, the HC diet significantly increased the concentrations of alkaline phosphatase (ALP) and amylase (AMY) in the blood and rumen fluid of goats (*p* < 0.05). Microbial analysis in the rumen of goats revealed that the HC diet decreased bacterial richness and diversity, as evidenced by the changed observed species, Chao 1, PD whole tree and Shannon when compared to the NC diet (*p* < 0.01). The proportion of Proteobacteria increased while that of Spirochaetes and Fibrobacteres significantly decreased with the HC diet (*p* < 0.05). The Christensenellaceae_R-7_group and Ruminococcaceae_UCG-010 in rumen was notably decreased when a diet was switched from 55% concentrate diet to 90% concentrate diet (*p* < 0.05). Additionally, microbial functional potentials deduced that the HC diet significantly increased the abundance of the citrate cycle (TCA cycle) (ko00020) associated with carbohydrate metabolism (*p* = 0.028). Furthermore, the HC diet significantly increased the glutathione metabolism (ko00480) associated with the metabolism of other amino acids (*p* = 0.008). Our findings suggested that SARA reduced the total antioxidant capacity and increased levels of inflammatory factors in goats, as well as decreased rumen bacterial species and abundance.

## 1. Introduction

In recent years, in pursuit of enhanced production efficiency, intensive farming has witnessed the widespread adoption of high-concentrate diets, primarily reliant on cereal starch as the primary energy source. Unfortunately, this extensive use has led to an upsurge in the prevalence of metabolic diseases. SARA is a prevalent gastrointestinal ailment in the field of ruminant farming. It is caused by excessive feeding of highly fermentable carbohydrates and insufficient dietary crude fiber [1]. A distinctive feature of SARA is the prolonged period of low rumen pH, with the rate of absorption and metabolism of rumen VFA serving as a pivotal determinant of rumen pH [2]. During the initial stages of SARA, typical symptoms are often absent, making diagnosis challenging. The primary diagnostic criterion for SARA is a rumen fluid pH persisting between 5.2 and 5.6 for over 3 h [3]. Consequences of SARA encompass reduced feed intake, diminished fiber digestibility, lower milk fat content, heightened occurrence of diarrhea, hoof inflammation, liver abscesses, elevated bacterial endotoxin levels, and increased acute phase protein during inflammation [4]. Owing to the scarcity of high-quality roughage resources in China, often characterized by subpar quality, producers find themselves compelled to heavily rely on starch-rich grain concentrates to fulfill the nutritional demands of animals, all in the pursuit of elevated production performance. Consequently, animals suffer from inadequate consumption of high-quality fiber, leading to the onset of acidosis, particularly prevalent in high-yield dairy cows and intensively fattened cattle and sheep. The emergence of SARA has emerged as a significant impediment to the production of cattle and sheep in China.

Research has demonstrated that in response to acid stress, microorganisms possess the ability to mitigate cell damage by modulating the dynamic equilibrium of intracellular pH (pHi), inducing the expression of stress proteins, and regulating cell membrane morphology and function [5]. Nevertheless, if stress surpasses the self-regulating capacity of microorganisms, intracellular protons and acid ions accumulate gradually, leading to a rapid decline in pHi. This, in turn, causes damage to cell membranes and internal macromolecular structures, disrupting normal cell growth and metabolism, and, in severe cases, resulting in microbial mortality [6,7,8]. The alterations in physical and chemical properties, reduced proliferation, and potential mortality of rumen microorganisms under acid stress constitute significant factors influencing rumen fermentation and shifts in microbial flora. Mertens [9] defined peNDF as: neutral detergent fiber (NDF) in the diet that can promote rumen liquid and solid phase stratification and affect the chewing of ruminants. Previous studies have shown that increasing the peNDF content of the diet increased the average rumen pH and is an effective means of preventing SARA [10,11].

As sequencing technology continues to advance, we have the opportunity to delve deeper into the influence of dietary changes on the gut microbiome of animals [12,13,14,15]. Maintaining relatively high hay feeding and a stable bacterial composition is imperative for the well-being of ruminants. SARA, on the other hand, leads to significant alterations in both rumen and intestinal microbiota [16,17,18]. Currently, research into the gut microbiota of both animals and humans is advancing, unveiling ever-expanding functions of the gut microbiota. Simultaneously, there is a continuous emergence of developments in gut microbiota preparations [19,20]. This experiment aims to construct a subacute rumen acidosis model in goats and study the changes in antioxidant capacity, immune levels, and microbial community structure and composition under sub healthy rumen conditions, enriching the survival and reproduction patterns of rumen bacteria under diseases, and providing a basis for the development of gut microbiota preparations for ruminants.

## 2. Materials and Methods

### 2.1. Animals and Treatment

This study was approved by the Animal Care and Use Committee of Hunan Agricultural University (HAU201408). To conduct this study, we chose sixteen Liuyang black goats at random. These goats were all six months old and had an average weight of 15.3 ± 1.67 kg. The goats were subsequently separated into two categories and given either a regular-concentrate meal (NC, with a ratio of 55% concentrate to 45% forage) or a concentrated diet (HC, with a ratio of 90% concentrate to 10% forage). Before the diets were formulated, the paddy straw was chopped to approximately 2 cm in length. The dry matter intake in the two groups was 572 g/d (NC) and 602 g/d (HC), respectively. Table 1 provided a comprehensive breakdown of the diets’ composition and nutritional value. The experimental period spanned 35 days, including a 7-day diet adaptation phase. The diets were evenly distributed to the goats at approximately 08:00 and 18:00 h, respectively. Each goat was housed in its individual enclosure.

### 2.2. Sample Collection

Upon completion of the 35-day feeding period, plasma samples were collected into tubes using the methodology described in a prior study [21], and subsequently stored at −20 °C for later analysis. Rumen epithelial samples were extracted and preserved in 10% neutral formalin to facilitate histomorphology examinations. Six animals were randomly chosen from each group and humanely euthanized. Rumen fluid was collected, promptly frozen at −80 °C, and later utilized for genomic DNA isolation and the assessment of enzyme metabolism indicators. The pH values in rumen samples of two groups in SARA model were the same before feeding in the morning (0 h), but it was declined in the HC group at 3 h or 6 h after feeding. During the time period from 3 h after the feeding until the sampling at 6 h, the average ruminal pH was below 5.7 in the HC group and remained significantly lower in NC goats [22]. Rumen segments from each goat were gathered to observe the morphology of the rumen epithelium.

### 2.3. Measurement of Enzyme Metabolism, Antioxidant and Immune Indicators

The T-AOC, NO, IFN-γ, HIS, IL-1β, IL-2, IL-4, and IL-6 in the blood were detected using a corresponding enzyme-linked immunosorbent assay (ELISA) kit (Jiangsu Yutong Biological Technology Co., Ltd. Yancheng, China). The blood and rumen fluid metabolites including ALT (alanine aminotransferase), AST (aspartate aminotransferase), ALP (alkaline phosphatase), and AMY (amylase) were detected with the commercial assay kits (Roche Diagnostics (Shanghai) Ltd., Shanghai, China; and determined using an Automatic Biochemistry analyzer (Cobas c 311, Roche, Shanghai, China), and strictly following the manufacturer’s instructions, respectively.

### 2.4. Histological Examination

Morphological observation involved the collection of rumen epithelial segments from every goat. The rumen epithelial tissues were dehydrated, embedded, and subsequently sliced into approximately 5 mm-thick sections using a microtome. Afterwards, they were dyed with hematoxylin and eosin. Images of the rumen epithelium’s structure were taken using a light microscope (Pannoramic DESK, P-MIDI, P250, 3DHISTECH, Budapest, Hungary) that had a computer-assisted morphometric system.

### 2.5. DNA Extraction and PCR Amplification

DNA extraction from rumen fluid was carried out using the E.Z.N.A.^®^ soil DNA Kit (Omega Bio-Tek, Norcross, GA, USA) and verified through 1% agarose gel electrophoresis [23]. The V3–V4 hypervariable regions of the bacterial 16S-rDNA gene were subsequently amplified using universal primers 338F (5′-ACTCCTACGGGAGGCAGCAG-3′) and 806R (5′-GGACTACHVGGGTWTCTAAT-3′). PCR reactions were conducted in a total volume of 20 μL, comprising 1 × FastPfu Buffer, 250 μM dNTPs, 0.1 μM of each primer, 1 U FastPfu Polymerase (Beijing TransGen Biotech, Beijing, China), and 10 ng of template DNA. The PCR protocol consisted of an initial denaturation at 95 °C for 2 min, followed by 30 cycles of denaturation at 95 °C for 30 s, annealing at 55 °C for 30 s, elongation at 72 °C for 30 s, and a final extension at 72 °C for 5 min. Subsequently, the amplification products were confirmed using 2% agarose gel electrophoresis. Purified amplicons were equimolarly pooled and subjected to paired-end sequencing (2 × 300) on an Illumina MiSeq platform (Allwegene, Beijing, China) following standard protocols [24].

### 2.6. Bacterial Data Processing and Function Predication

The FASTQ files were processed using QIIME (version 1.17) by demultiplexing and quality filtering. The following criteria were applied: (i) Reads with a length of 300 bp were truncated at positions where the average quality score dropped below 20 within a 10 bp sliding window. Reads shorter than 50 bp after truncation were discarded. (ii) Stringent processing involved precise barcode matching, allowing for a maximum of two nucleotide mismatches in primer matching, and excluding reads containing ambiguous characters. (iii) Assembly of sequences was performed only for those with overlaps longer than 10 bp in their overlapping regions. The unassembled reads were eliminated. OTUs were clustered using UPARSE (version 7.1) with a 97% similarity cutoff, and chimeric sequences were identified and removed using UCHIME (https://www.drive5.com/uchime/uchime_download.html, accessed on 8 January 2024). A rarefaction analysis was performed using Mothur v.1.21.1 to evaluate diversity indices including observed_species, PD_whole_tree, and Shannon. Primer 6 software (Primer-E Ltd., London, UK) was used for conducting hierarchical clustering analysis, and Canoco 4.5 was employed for performing Principal Component Analysis (PCA). The bioinformatics tool PICRUSt was used to predict the functional potentials of metagenomes by reconstructing unobserved states through a phylogenetic investigation of communities, relying on data from the 16S rRNA gene [25]. The OTU table was utilized in PICRUSt to predict functional genes, making use of the Kyoto Encyclopedia of Genes and Genomes (KEGG) database.

### 2.7. Statistical Analysis

All of the experimental data in the tables and figures are presented as the mean ± SD and the differences between groups were assessed by the independent-samples *t* test using SPSS (IBM SPSS 21.0, Chicago, IL, USA). The data of 16S rRNA sequencing were analyzed on the allwegene platform. Statistical significance was set at *p* < 0.05 and tendencies at 0.05 ≤ *p* ≤ 0.10.

## 3. Results

### 3.1. Antioxidant and Immune Indicators of Growing Goats Fed Different Diets

**Figure 1:**
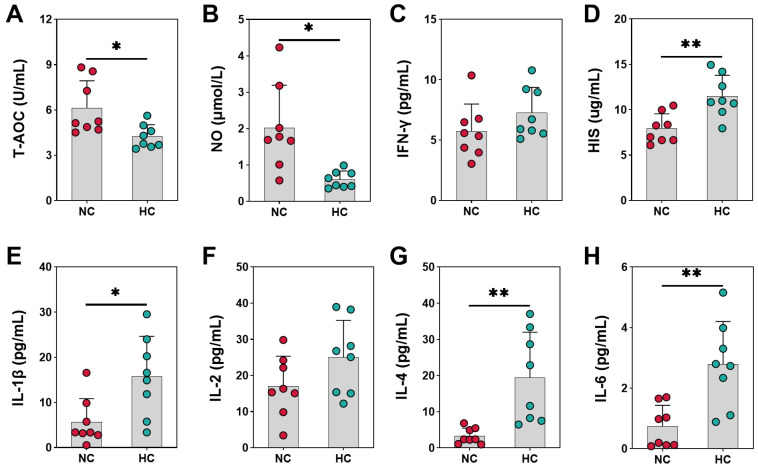
The antioxidant and immune indicators of growing goats fed normal-concentrate diet (NC) and high-concentrate diet (HC). (**A**) T-AOC, total antioxidant capacity; (**B**) NO, Nitric Oxide; (**C**) IFN-γ, interferon-γ; (**D**) HIS, Histamine; (**E**) IL-1β, interleukin-1β; (**F**) IL-2, interleukin-2; (**G**) IL-4, interleukin-4; (**H**) IL-6, interleukin-6. (*) 0.01 < *p* < 0.05; (**) *p* < 0.01.

As shown in Figure 1A, the T-AOC was decreased dramatically in plasma by feeding the HC diet to goats compared to the NC diet (*p* = 0.022). Compared with the NC group, the data also showed that the NO level of goats was improved notably by the HC diet (*p* = 0.011) (Figure 1B). And diets supplemented with the HC diet has no significant effect on the IFN-γ (Figure 1C), there were no statistical differences between the groups (*p* = 0.185). The HC diet raised the HIS concentration of goats remarkably (*p* = 0.003) (Figure 1D). Results for plasma IL-1β was significantly boosted for the HC group compared to the NC group (*p* = 0.015) (Figure 1E). The level of IL-4 (*p* = 0.008) and IL-6 (*p* = 0.002) in plasma increased significantly when dietary concentrate ratio increased from 55% to 90% (Figure 1G,H). Compared with the NC group, plasma concentration of IL-2 in the HC group was not significantly different compared to the NC group (*p* = 0.110) (Figure 1F).

### 3.2. The Enzyme Metabolism Indicators of Growing Goats Fed Different Diets

**Figure 2:**
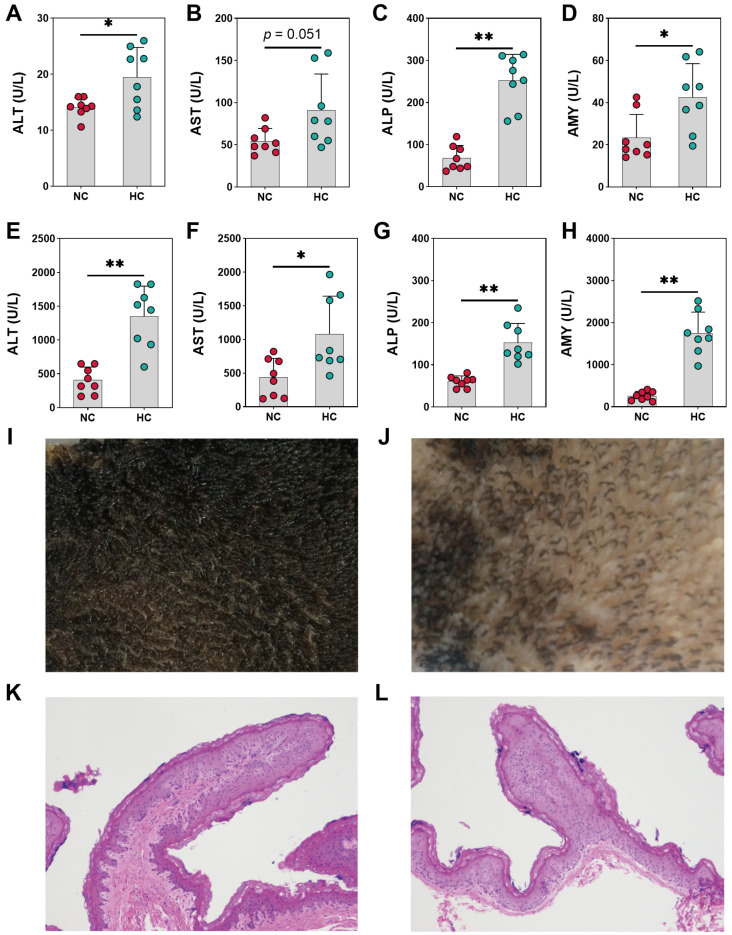
The enzyme metabolism indicators of growing goats fed normal-concentrate diet (NC) and high-concentrate diet (HC). (**A**–**D**) The enzyme metabolism indicators in plasma. (**E**–**H**) The enzyme metabolism indicators in rumen. The representative original images (**I**,**J**) and light micrographs (**K**,**L**) of ruminal epithelium in goats fed the NC and HC diets are shown. (*) 0.01 < *p* < 0.05; (**) *p* < 0.01.

The results shown in Figure 2A demonstrated that the ALT concentration in plasma was increased by the HC diet (*p* = 0.025). And the AST concentration in plasma was also increased by the HC diet compared to the NC diet (*p* = 0.051) (Figure 2B). The ALP concentration in plasma was notably increased when a diet administered to steers was switched from 55% concentrate diet to 90% concentrate diet (*p* < 0.001) (Figure 2C). Results for plasma AMY concentration were significantly boosted for the HC group compared to the NC group (*p* = 0.015) (Figure 2D), where ALT (*p* < 0.001) and AST (*p* = 0.025) concentrations in rumen were increased in this experimental model by increasing the grain percentage of the diet to induce SARA (Figure 2E,F). And the HC diet elevated significantly the ALP (*p* < 0.001) and AMY (*p* < 0.001) in rumen compared to the NC diet fed to growing goats (Figure 2G,H). The rumen epithelium from the NC group showed an intact structure while the rumen epithelium of the HC group showed an indication of sloughing in the stratum corneum (Figure 2I–L).

### 3.3. Rumen Microflora of Growing Goats Fed Different Diets

**Figure 3:**
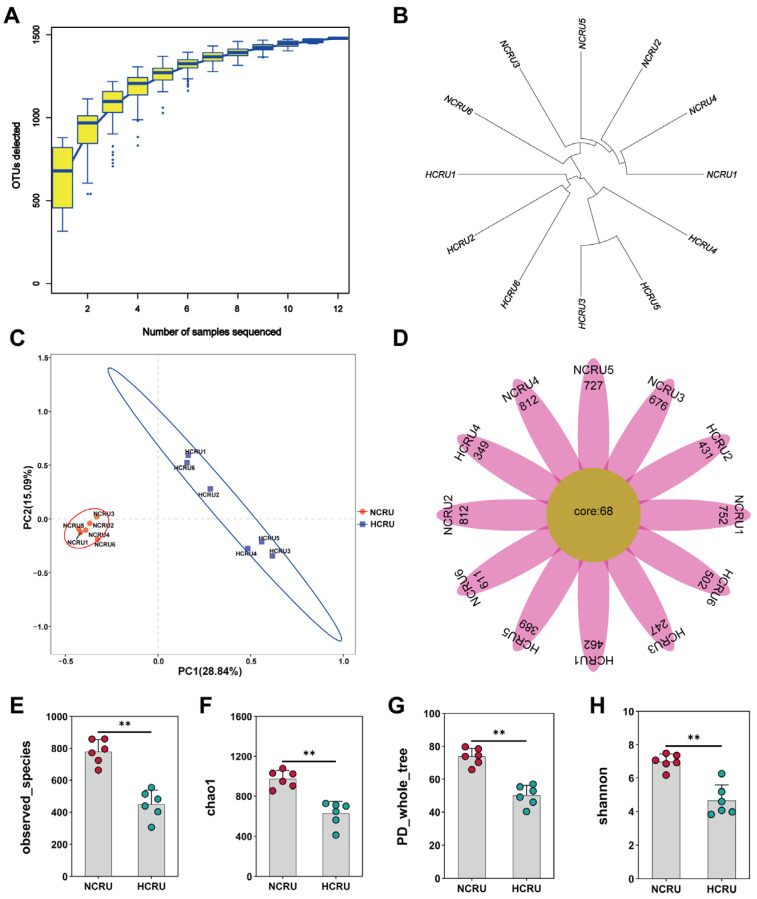
Rumen microflora of growing goats fed normal-concentrate diet (NC) and high-concentrate diet (HC). (**A**) Species accumulation curves. (**B**) Hierarchical clustering (NCRU1–NCRU6 and HCRU1–HCRU6 are rumen samples of goats fed with 55% or 90% concentrate, separately). (**C**) Principal component analysis (PCA) of rumen bacterial community. (**D**) Flower plot. (**E**–**H**) Alpha diversity indices of rumen bacterial community. (**) *p* < 0.01.

The increase in species as the sample size increases was described using species accumulation curves (Figure 3A). The findings indicated the frequency at which novel OTUs (novel species) emerge through ongoing sampling. In a specific interval, when the sample size grows, a steep rise in the curve suggests the presence of numerous species in the community. Conversely, a flattening curve indicates that the species in this habitat do not substantially increase as the sample size increases. Our results indicated that sampling is sufficient for microbiological data analysis. Based on the analysis of hierarchical clustering, the branch length indicates the distance between samples, and samples with greater similarity tend to cluster closer together from Figure 3B. Based on the evidence, it can be verified that the rumen samples from goats that were given regular and high-concentrate diets were grouped distinctly. The PCoA revealed distinct separation between rumen samples in the NC group and those in the HC group, as demonstrated in Figure 3C. The flower plot represents a sample with each petal, with the core number in the middle representing the number of OTUs common to all samples, and the number on the petal representing the unique number of OTUs for that sample. it can be proven that the HC diet reduces the number of OTUs in the rumen compared to the NC diet fed to growing goats (Figure 3D). The observed species was markedly lower in the HC group than in the NC group (*p* < 0.05) (Figure 3E). In the rumen fluid, the Chao 1, PD whole tree, and the Shannon index notably decreased (*p* < 0.01 for Chao 1 and PD whole tree, *p* < 0.05 for the Shannon index) when the goats’ diet was changed from 55% concentrate to 90% concentrate (Figure 3F–H). Hence, the HC diet declined the rumen bacteria richness and diversity.

### 3.4. Rumen Bacterial Community Structure of Growing Goats Fed Different Diets

**Figure 4:**
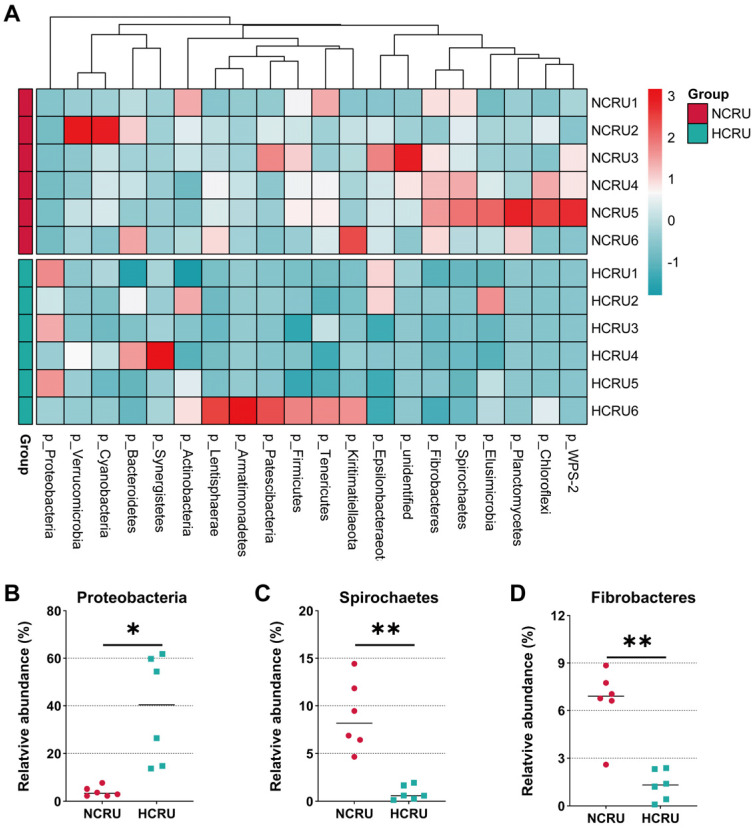
(**A**) Heatmap based on hierarchical clustering solution at phylum level (Bray-Curtis distance metric and complete clustering method) in rumen for goats fed a normal diet (NCRU) or a high-concentrate diet (HCRU), shown across columns. (**B**–**D**) Differential bacteria in the rumen at the phylum level. (*) 0.01 < *p* < 0.05; (**) *p* < 0.01.

Bacteroidetes, Proteobacteria and Firmicutes were dominant phyla in the rumen of goats, accounting for more than 87% of the total rumen bacterial community (Figure 4A). Bacteroidetes and Proteobacteria accounted for a relative abundance of 45.9% and 21.3%, respectively, followed by Firmicutes, at 20.7%. When the diet of concentrate proportion increased from 55% to 90%, the abundance of Proteobacteria was increased significantly in the HCRU group than the NCRU group (*p* < 0.05) (Figure 4B). However, the proportion of Spirochaetes decreased dramatically in goats when the diet administered to goats was switched from 55% concentrate diet to 90% concentrate diet (*p* < 0.01) (Figure 4C). At the same time, the HC diet significantly dropped the abundance of Fibrobacteres (*p* < 0.01) (Figure 4D).

**Figure 5:**
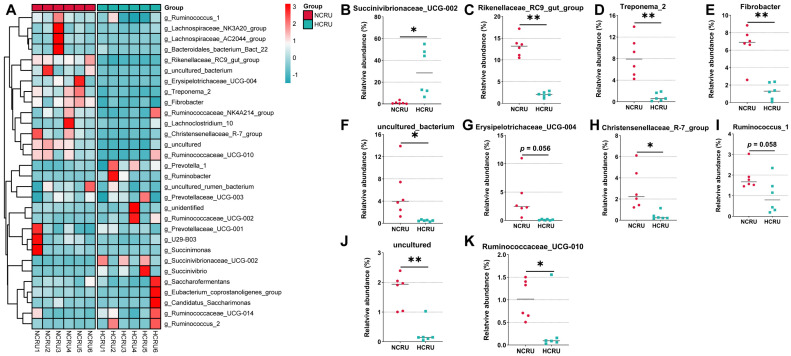
(**A**) Heatmap based on hierarchical clustering solution at genus level (Bray-Curtis distance metric and complete clustering method) in rumen for goats fed a normal diet (NCRU) or a high-concentrate diet (HCRU), shown across rows. (**B**–**K**) Differential bacteria in the rumen at the genus level. (*) 0.01 < *p* < 0.05; (**) *p* < 0.01.

Within the bacterial population, the top 30 genera were identified across all samples (Figure 5A)-the genus Succinivibrionaceae_UCG-002 was the most abundant genera, accounting for 15.4%, followed by Prevotella_1, Rikenellaceae_RC9_gut_group, Treponema_2, and Fibrobacter as the predominant genera of rumen in the NCRU and HCRU groups. Among the genera, the percentage abundance of Succinivibrionaceae_UCG-002 was greater in the rumen of the HCRU group when compared to that in the NCRU group (*p* < 0.05) (Figure 5B), and Erysipelotrichaceae_UCG-004 tended to be lower by the HC diet (*p* = 0.056) (Figure 5G). In contrast, the relative abundance of Rikenellaceae_RC9_gut_group, Treponema_2 and Fibrobacter was markedly decreased in the rumen of the HCRU group than in the NCRU group (*p* < 0.01) (Figure 5C–E), and Ruminococcus_1 tended to decrease by the HC diet (*p* = 0.058) (Figure 5I). The Christensenellaceae_R-7_group and Ruminococcaceae_UCG-010 in rumen was notably decreased when a diet administered to steers was switched from 55% concentrate diet to 90% concentrate diet (*p* < 0.05) (Figure 5H,K).

### 3.5. Function Prediction of Rumen Bacterial Community Using PICRUSt

**Figure 6:**
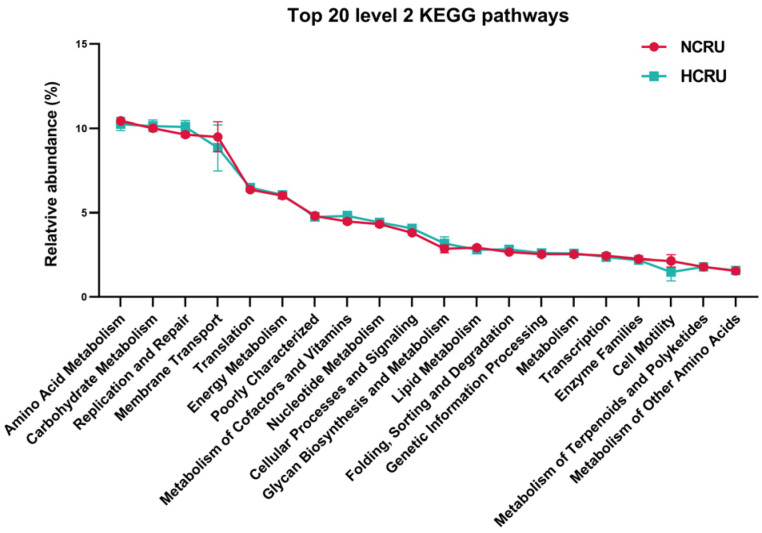
Top 20 predicted metagenomic functions at level 2 of the KEGG (Kyoto Encyclopedia of Genes and Genomes) pathways in rumen bacteria of goats fed the NC and HC diets.

Functional potentials of rumen microbiota were predicted using 16S rRNA marker gene sequences through PICRUSt against KEGG pathways. As showed in Figure 6, within the 20 most abundant level 2 KEGG pathways, ten, amino acid metabolism, carbohydrate metabolism, energy metabolism, the metabolism of cofactors and vitamins, nucleotide metabolism, glycan biosynthesis and metabolism, lipid metabolism, enzyme families, the metabolism of terpenoids and polyketides, and the metabolism of other amino acids, were associated with metabolism; and one, membrane transport, was associated with environmental information processing; four, including replication and repair, translation, folding, sorting and degradation, and transcription were associated with genetic information processing; one, cell motility was associated with cellular processes. 

In total, the related amino acid metabolism, carbohydrate metabolism and the metabolism of other amino acids individual pathways were predicted, and a comparison of different pathways (as shown in Table 2) revealed that the HC diet influenced the functional potentials of rumen microbiota. Specifically, compared to the NC group, the HC diet tended to decrease the abundance of histidine metabolism (ko00340) (*p* = 0.081), and the HC diet significantly decreased the abundance of lysine degradation (ko00310), tryptophan metabolism (ko00380), valine, leucine and isoleucine degradation (ko00280), and selenocompound metabolism (ko00450) (*p* < 0.05). In contrast, the HC diet significantly increased the abundance of the citrate cycle (TCA cycle) (ko00020) associated with carbohydrate metabolism (*p* = 0.028). Furthermore, the HC diet significantly increased the glutathione metabolism (ko00480) associated with the metabolism of other amino acids (*p* = 0.008). 

## 4. Discussion

The diet’s formulation and composition can potentially impact the growth performance and antioxidant capacity of animals, as stated in references [26,27,28]. Zhang et al. showed that SARA elevated systematic oxidative status and enhanced autophagy in the liver, and suppressed SIRT1 and FOXA2 may mediate enhanced oxidative damage and autophagy in the livers of dairy cows fed a HC diet [29]. Cows with SARA often develop complications or other diseases with physiologic immunosuppression and inflammation [30]. Our current study has verified that the HC diet could diminish the antioxidant capacity of goats, underscoring the potential adverse consequences of indiscriminately promoting weight gain in these animals. Dietary patterns can impact not only body weight but also bone density [31]. In addition to antioxidant capacity, anti-inflammatory and immunomodulatory properties are equally vital for the well-being of the host [32]. Cytokines, produced and released by immune cells that are activated, constitute a category of biologically active substances. Immune cells interact and mutually regulate each other, playing crucial parts in inflammation, immune responses, tissue healing, and hematopoietic functions. Promoting the proliferation of immune cells are several noteworthy immune mediators, namely IL-2, IL-4, IL-6, IL-10, and IL-12 [33,34,35]. Feeding goats with a high-concentrate (HC) diet resulted in lower nitric oxide (NO) production compared to the normal group. Typically, increased NO levels are associated with an enhanced pro-inflammatory response to lipopolysaccharides (LPS) [36], which correlates with elevated concentrations of various interleukins. Prior research has demonstrated that elevated IL-6 levels may enhance the permeability of the rumen epithelium and diminish its barrier function [37]. Furthermore, our research validated that the nitric oxide (NO) [38] mediated the control of IL-2 and IL-6 production in these goats through a negative feedback mechanism. These findings suggest that feeding goats with a high-concentrate (HC) diet during a state of subacute rumen acidosis (SARA) results in elevated plasma NO levels, which subsequently suppresses the production of cytokines, notably IL-6.

The activity of animal digestive enzymes [39] can be significantly influenced by the composition of the diet. The digestive enzymes produced by rumen microorganisms in ruminants help to break down and utilize the natural polymers found in their feed [40]. The high-carbohydrate diet resulted in an increase in AMY activity, in line with previous studies suggesting that higher intake of starch leads to elevated AMY activity in the contents of the pancreas and small intestine [41,42]. ALT and AST activity are reliable markers of liver function, released into the bloodstream when the liver is compromised. Typically, serum AST activity remains low under normal conditions but significantly elevates in the presence of cell damage. Increased AST and ALT values are indicative of varying degrees of liver impairment [43]. The HC group displayed elevated AST and ALT values in contrast to the NC group, implying that the HC diet may lead to liver damage in goats. ALP is an enzyme widely distributed in tissues and organs, including the liver, bones, and small intestine. It facilitates the hydrolysis of phosphate into phosphoric acid under alkaline conditions. Various forms of ALP, including small intestine ALP and bone-type ALP, contribute to the regulation of intestinal function, as well as calcium and phosphorus metabolism [44,45]. DiLorenzo and McCarthy, among others, have provided evidence suggesting that AMY may potentially accelerate the rate of ruminal carbohydrate degradation [46,47]. Dietary nutrients have the capacity to impact the morphology of small intestinal tissue and the digestive function of animals [48]. An imbalance in the interplay between gut microbiota and other factors can disrupt the homeostasis of the intestinal mucosa [49]. The intestinal epithelial barrier, acting as the first line of defense between the luminal environment and the host, can result in severe inflammation or other intestinal diseases if compromised [50]. This study additionally furnishes evidence that diets can impact the morphology of the rumen epithelium. Elevated enzyme levels (ALT, AST, and ALP) indicate potential damage or alterations in membrane permeability [51]. As a result, feeding goats an HC diet may have detrimental effects on their intestinal epithelium.

For this research, we employed 16S rRNA sequencing to investigate the bacteria present in rumen fluid. Our focus was on observing the changes in the composition and structure of rumen flora as goats progressed from a state of good health to one of acidosis. Our goal was to explore the reproduction patterns of rumen bacteria during disease and enhance the understanding of rumen microecology. Species accumulation curves are useful instruments for analyzing the species makeup of samples and forecasting species abundance. In biodiversity and community surveys, they are extensively used to assess the adequacy of sample size and estimate the diversity of species [52]. Thus, species accumulation curves not only assess sample size sufficiency but also predict species richness when the sample size is adequate. Utilizing the beta diversity distance matrix for hierarchical clustering analysis [53], the tree structure was constructed using the UPGMA algorithm, which helped with visual analysis. Our research validates the appropriateness of sequencing rumen samples from goats subjected to the NC and HC diets, revealing distinct clustering patterns for the two treatment groups. Rumen environmental imbalances often result in shifts in rumen flora. For example, Kim et al. induced subacute rumen acidosis (SARA) through a high-grain diet, which led to decreased rumen flora diversity and a reduction in the relative abundance of Prevotella [54]. Our study reveals that goats fed a HC diet display lower bacterial richness and diversity compared to those fed the NC diet, as evident from reductions in observed species, Shannon, Chao 1, and PD whole tree indices. This observation highlights the notably detrimental effect of the HC diet on the biodiversity of the goat rumen ecosystem. 

Modifying dietary formulations can lead to shifts in the microbial composition of the animal gut and impact digestive function [55,56]. Zhao et al. indicated the importance of bacterial sphingolipids in maintaining hindgut symbiosis and homeostasis. Dietary supplementation with citrus flavonoid extract can decrease systemic inflammation by maintaining hindgut microbiota homeostasis and regulating sphingolipid metabolism in dairy cows fed a high-starch diet [57]. Environmental acid stress has a substantial impact on the growth and reproduction of microorganisms during microbial fermentation [58,59]. Previous studies have reported that Bacteroides is a major anaerobic genus in the rumen [60], and high-concentrate diets have been associated with a decrease in Bacteroides populations [61,62]. Prevotella, a member of the Bacteroides genus, is the predominant genus of rumen bacteria [63]. Prevotella is known for its high activity in protein degradation and its ability to utilize starch and pectin [60]. Surprisingly, our findings revealed no notable alteration in the prevalence of Prevotella in the rumen prior to and following SARA, which contradicts earlier research. Considering our experimental design, it is hypothesized that Prevotella may rapidly multiply when provided with ample starch substrates in a short timeframe, maintaining a stable relative abundance. The health and function of the rumen greatly depend on the diversity of microorganisms. During our investigation, we noticed a decrease in the ratios of the Rikenellaceae RC9 intestinal cluster, Treponema 2, Fibrobacter, and Christensenellaceae R-7 cluster when exposed to high-calorie feeding circumstances. It is worth mentioning that Treponema and Fibrobacter are acknowledged for their role in breaking down fibers [64,65]. Numerous studies on SARA have extensively documented the abundance of bacteria that break down starch and the decrease in bacteria that break down fiber [66,67]. Our current research aligns with these findings, confirming disruptions in rumen digestion and metabolic capabilities in the HC-fed goats. Succinivibrionaceae UCG-002 is a member of the Succinivibrionaceae family and has been associated with ruminal fatty acid metabolism [68]. As for the Rikenellaceae RC9 gut cluster, the Rikenellaceae family is a fairly recent taxonomic categorization, and there is a significant requirement for additional information regarding its metabolic role, despite the research conducted by Su et al.suggested a single isolate that produces acetate from the Rikenellaceae family [69]. Rikenellaceae RC9 was able to alter the digestibility of dietary NDF and ADF as well as the VFA concentration, as shown by Zhao et al. [70]. The Christensenellaceae R-7 cluster is most closely associated with Christensenella minuta [71], a species capable of generating acetate and butyrate through glucose [72]. In summary, these findings indicated that the HC goats may have impaired rumen digestion and metabolic capabilities.

Rumen bacteria play a crucial role in maintaining ruminant health [73,74,75]. In our study, we used PICRUSt to assess the potential functions of bacteria associated with digestion in the rumen. The clear disparities in anticipated functional pathways between the NC and HC groups suggest that alterations in bacterial populations can influence the functional potential of the rumen bacterial community. Within the HC group, we noticed a decline in pathways associated with the metabolism of amino acids, such as histidine, lysine, tryptophan, valine, leucine, and isoleucine breakdown. This indicates possible detrimental impacts on the metabolism of amino acids in the host resulting from prolonged consumption of an HC diet. Additionally, we noted a relative increase in pathways associated with the tricarboxylic acid (TCA) cycle and carbohydrate metabolism in the HC group, suggesting that the rumen microbiomes of HC-fed goats may have an enhanced capacity for energy extraction. Overall, these predicted findings suggest that long-term HC diet feeding may impact the bacterial function of the rumen microbiome in goats. However, it is important to note that while PICRUSt is a well-validated predictive tool, true metagenomic shotgun sequencing is necessary for a more comprehensive examination of the effects of HC diets on rumen-associated bacteria in goats.

## 5. Conclusions

In summary, SARA could alter the total antioxidant capacity and inflammatory factors levels of goats, and affect enzyme metabolism in their blood and rumen. Through our research, we have gained a deeper comprehension of the microorganisms present in the rumen, the presence of SARA reduced the number and variety of bacteria in the rumen and may affect the development and performance of bacteria in the rumen.

## Figures and Tables

**Table 1:** Ingredients and nutrient levels of the experimental diets (air-dried basis).

Item	NC ^1^	HC ^2^
Ingredients composition (%)		
Forage		
Rice straw	45.0	10.0
Concentrate		
Rice with shell	33.2	54.3
Soybean meal	9.60	15.7
Wheat bran	6.00	9.80
Fat powder	3.20	5.20
Calcium carbonate	0.50	0.80
Calcium bicarbonate	1.10	1.80
Sodium chloride	0.60	1.00
Premix ^3^	1.00	1.40
Nutrient levels ^4^, % of DM		
Crude protein	13.5	17.6
Crude ash	9.34	9.12
Crude fat	4.18	6.01
Neutral detergent fiber	49.8	38.4
Acid detergent fiber	36.5	9.51
Starch	26.57	38.57

^1^ NC: normal-concentrate diet. ^2^ HC: high-concentrate diet. ^3^ Premix composition per kg diet: 68 mg FeSO_4_·H_2_O, 44 mg CuSO_4_·5H_2_O, 411 µg CoCl_2_·6H_2_O, 1.70 mg KIO_3_, 211 mg MnSO_4_·H_2_O, 126 mg ZnSO_4_·H_2_O, 56 µg Na_2_SeO_3_, 462 mg MgSO_4_·7H_2_O, 737 IU vitamin A, 8.29 mg vitamin E, 5.1 g carrier zeolite powder. ^4^ Nutrient levels were measured values.

**Table 2:** KEGG pathways that showed different abundances between ruminal digesta microbiota of NC- and HC-diet goats.

Level 2	Level 3	Pathway ID	NC ^1^	HC ^2^	*p*-Value
Amino acid metabolism	Histidine metabolism	ko00340	0.69 ± 0.03	0.65 ± 0.05	0.081
	Lysine degradation	ko00310	0.16 ± 0.02	0.13 ± 0.02	0.030
	Tryptophan metabolism	ko00380	0.19 ± 0.01	0.16 ± 0.02	0.003
	Valine, leucine and isoleucine degradation	ko00280	0.29 ± 0.02	0.25 ± 0.02	0.006
Carbohydrate metabolism	Citrate cycle (TCA cycle)	ko00020	0.73 ± 0.05	0.80 ± 0.04	0.028
Metabolism of other amino acids	Glutathione metabolism	ko00480	0.17 ± 0.004	0.24 ± 0.05	0.008
	Selenocompound metabolism	ko00450	0.38 ± 0.01	0.34 ± 0.02	0.002

^1^ NC: normal-concentrate diet; ^2^ HC: high-concentrate diet.

## Data Availability

The data presented in this study are available on request from the corresponding author. The availability of the data is restricted to investigators based in academic institutions.
